# Supplementary figures and images for: Comparative Proteomics and Interactome Analysis of the SARS-CoV-2 Nucleocapsid Protein in Human and Bat Cell Lines
Source: Viruses. 2024 Jul 11;16(7):1117. doi: 10.3390/v16071117 (PMC11281661; doi:10.3390/v16071117)

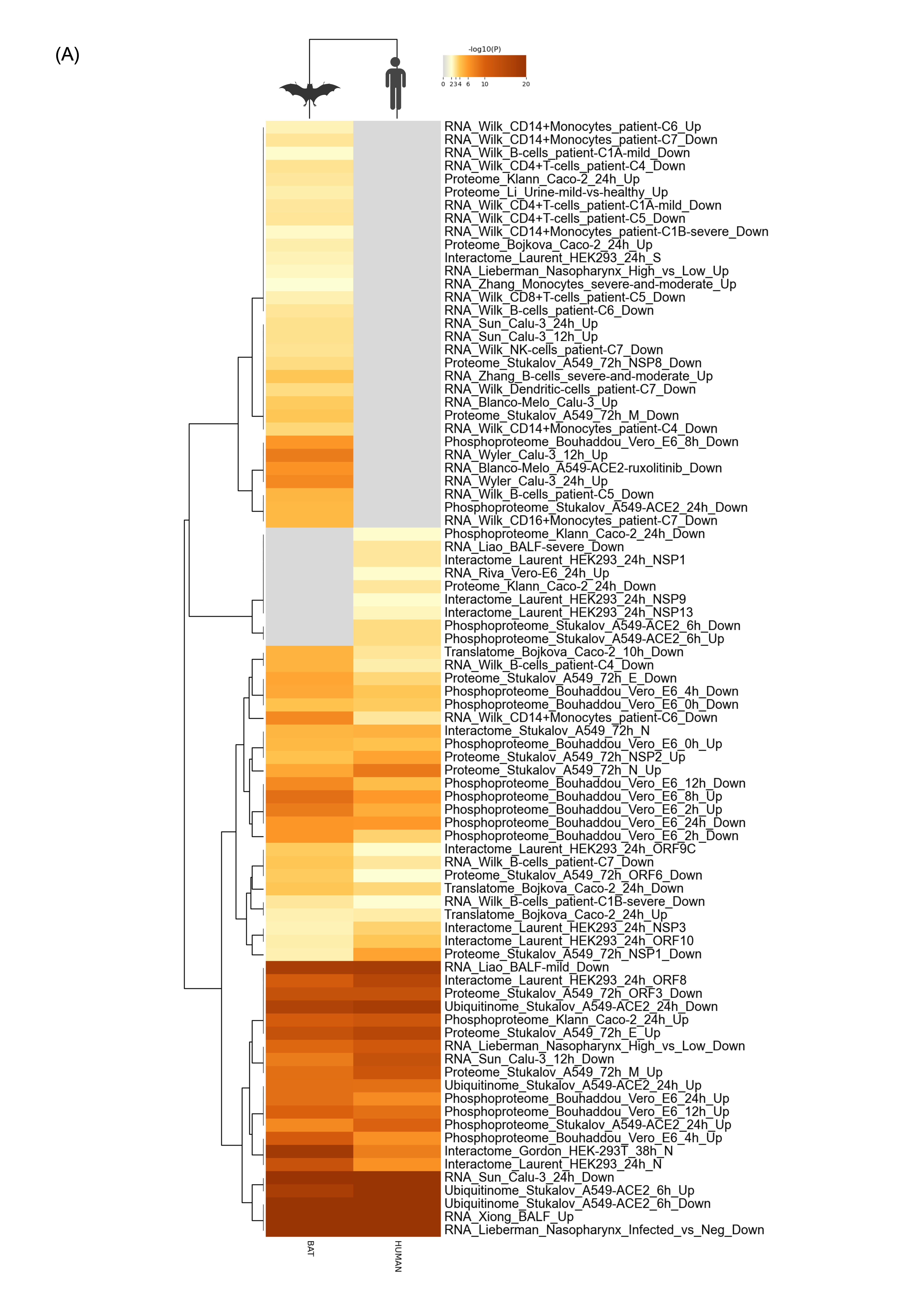

Supplement: Supplementary file 1 [file viruses-16-01117-s001.zip › FIGURE S2.png]
